# An examination of the relationship between risk perceptions, cultural-religious beliefs and coping during COVID-19 pandemic control in South Asian countries: a systematic review

**DOI:** 10.1186/s40359-024-01963-8

**Published:** 2024-08-31

**Authors:** Rakhshi Memon, Ayesha Khaliq, Veronica Ranieri, Muqaddas Asif, Mujeeb Masood Bhatti, Bilal Ahmad Khan, Nasim Chaudhry, Imran B. Chauhdry, Nusrat Husain, Sarah J. L. Edwards

**Affiliations:** 1 Department of Science, Technology, Engineering and Public Policy (STEaPP), University College London (UCL), London, UK; 2 Pakistan Institute of Living and Learning, Karachi, Pakistan; 3 University of York, York, UK; 4 Ziauddin University Hospital, Karachi, Pakistan; 5 The University of Manchester, Manchester, UK; 6 Mersey Care NHS Foundation Trust, Liverpool, UK; 7 DOW University of Health Sciences, Karachi, Pakistan

**Keywords:** Covid-19, Religious- cultural belief, KAP

## Abstract

**Background:**

Covid 19 was declared as a public health emergency by the World Health Organisation (WHO) due to its rapid spread and catastrophic effects on health. It affected around 119 M people with mortality rate of 0.27% worldwide, including South-Asians. This review aims to understand the risk perceptions, cultural religious beliefs and the coping mechanisms of South Asians during the Covid 19 pandemic.

**Methods:**

We conducted a systematic review following the Preferred Reporting Items for Systematic Reviews and Meta-Analyses (PRISMA) guidelines. The following search engines were used: Medline, Cochrane Library, PsycINFO, CINAHL, and Web of Science. Included studies investigated perceptions and opinions of individuals on knowledge, risk and protective factors, native faith based practices, and attitudes towards the COVID-19 pandemic.

**Results:**

The database search produced 282 articles to screen. The final narrative synthesis included five studies comprising of 13,476 participants from Pakistan, India, Nepal, and Bangladesh. Ten studies, comprising 7,893 participants, were eligible and included for meta-analysis. The overall pooled prevalence with maximum heterogeneity for correct knowledge of symptoms, hand washing or use of sanitizers, face masking use of herbal or traditional remedies and physical distancing or avoidance of contact was reported through meta-analysis.

**Conclusion:**

The review brings forth a useful comparison of individual and cultural differences in KAP, risk perceptions and coping strategies. This review highlights the need for and importance of tailored information dissemination, culturally sensitive risk communication, targeted educational interventions, community engagement and empowerment, policy, and infrastructure improvements, as well as continued research and data collection. By addressing these implications, efforts to mitigate the impact of COVID-19 can be more effective and equitable across diverse populations.

**Prospero registration:**

CRD42021246475.

## Introduction

A novel coronavirus SARS-Cov-2 was first identified as a causal pathogen of COVID-19 disease in humans in December 2019 in Wuhan, China [1]. COVID-19 spread rapidly around the globe and was declared a pandemic by the World Health Organisation (WHO) on March 11, 2020. Since it was first identified, SARS-CoV-2 has infected more than 119 M individuals around the globe (WHO, 2020) with a mortality rate of 0.27% [2]. South Asian countries account for 10% of COVID-19 cases around the globe [2] with a Case Fatality Rate (CFR) of 3.5% which is far less than that of economically developed countries (8.0%) [3, 4] owing to the differences in the structure of age group [5]. Statistical trends indicate that, among South Asian countries, India has the highest number of reported COVID-19 cases and deaths whilst Bhutan has the lowest [6].

Containment of COVID-19 is heavily dependent on the precautionary measures taken by the masses [7] which are, in turn, dependent on risk perception [8] and health beliefs [9]. Risk perception is a subjective judgement or belief of an individual regarding the severity of potential harm and an important driving factor of protective behavior [10, 11]. In addition to risk perception, health beliefs also play an important role in determining attitudes and behaviour towards the pandemic [12]. As per the Health Belief model, perceived benefits, perceived susceptibility, perceived severity, perceived barriers, and cue-to‐action influence such attitudes and behaviour [13].

In addition to risk perception and health beliefs, the ‘Knowledge, Attitude, and Practice’ (KAP) framework has also been used to identify how knowledge about diseases can affect attitude, practice, and disease burden associated with it [14–16]. In the context of COVID-19, KAP refers to understanding people’s correct knowledge about the virus, their attitudes towards it, and their native faith based practices they adopt to prevent its spread. Knowledge about COVID-19 is relatively high among the general population and they hold a positive attitude towards protective measures such as wearing a mask, washing hands, and using hand sanitizer etc. [17–19]. However, the most common source of knowledge about COVID-19 is social media [20, 21]. The KAP has been noted as above average among individuals with higher education, females, and healthcare professionals [22]. Similarly, females are more inclined towards taking precautionary measures than males [19, 20].

High-risk perception and perceived severity of COVID-19 can have a direct impact on the mental health of the individual [23–25]. Commonly experienced mental disorders during the COVID-19 pandemic in South Asian countries include nonpsychotic depression, anxiety, insomnia, alcohol-related disorder, and somatic concerns [23, 25]. however, due to the strict adherence of most South Asian countries to religion, most inhabitants of these countries tend to turn towards religion and use religious coping mechanisms to deal with major life stressors [26]. Religiously framed behavioral, emotional, or cognitive responses to stressors are known as religious coping [27]. In other words, religious coping refers to help-seeking from religion – holy scriptures and therapy from religious leaders – in a stressful situation to reduce distressing thoughts and emotions [28]. Religious coping during COVID-19 has shown evidence to lower depressive symptoms [29] and stress [30], lesser loneliness [31] improve positive affect and life satisfaction [32]. Though literature examining risk perceptions, cultural-religious beliefs and coping during the covid-19 pandemic from many countries is available, a combined glance especially through the lens of a multicultural and multi-religious group like South Asia remains understudied to date. For example, a review of pandemic perceptions [33] found that different religious traditions hold differing beliefs (it’s a religious curse or only religion can save us) regarding infectious diseases. Therefore, the current systematic review aims to find out the KAP of South Asians toward COVID-19 and their coping mechanism for dealing with COVID-19, Which focuses on.

1. Knowledge: This refers to what South Asians know about COVID-19. It could include their correct understanding of the virus, its transmission, symptoms, preventive measures, available treatments, and vaccination.
2. Attitudes: This encompasses the beliefs, opinions, and perceptions that South Asians hold about COVID-19. It could include their level of concern, fear, trust in authorities or healthcare systems, perception of risk, and attitudes towards preventive measures such as mask-wearing, social distancing, and vaccination.
3. Practices: This refers to the actions and behaviors that South Asians adopt in response to COVID-19. It could include their adherence to preventive measures, such as wearing masks, practicing hand hygiene, maintaining social distance, avoiding large gatherings, and seeking healthcare when necessary.

Moreover, the systematic review aims to explore the native faith based practices employed by South Asians to deal with the challenges posed by COVID-19. Coping mechanisms are the strategies individuals use to manage stress, anxiety, and other negative emotions associated with the pandemic. These mechanisms could include seeking social support, engaging in positive activities, practicing mindfulness or relaxation techniques, maintaining routines, and accessing mental health services.

## Method

### Protocol registration

A systematic review protocol was developed and registered online with PROSPERO (CRD42021246475). This review followed the Preferred Reporting Items for Systematic Reviews and Meta-Analyses (PRISMA) reporting guidelines [34].

### Databases and search strategy

The following electronic databases (Inception to 1st November 2020): Medline, Cochrane Library, PsycINFO, CINAHL, and Web of Science, were searched using four concepts including knowledge and practices, culture, COVID-19, and South Asia. The overall search strings were: (Perception OR Knowledge OR Information OR Attitude* OR Awareness OR Practices OR Opinions OR Beliefs) AND (Religiou*) AND (COVID-19 OR COVID OR Coronavirus OR SARS-CoV-2) AND (Pakistan OR India OR Bangladesh OR Sri Lanka OR Nepal OR Bhutan OR Maldives OR Afghanistan OR South Asia*) (*See* Table 1). A search update was run from 2nd November 2020 to 28th Feb 2024 in get all other potential eligible articles.

### Eligibility criteria and selection of studies

This review looked for studies with any quantitative data including but not limited to cross-sectional, cohort studies, case-control studies, interrupted time series or mixed methods research. No restrictions were placed on participants’ characteristics about age, morbidity, or socio-economic status. Included studies investigating perceptions and opinions of individuals on knowledge, risk and protective factors, practices, cultural traditions, and attitudes towards the COVID-19 pandemic. Studies reporting findings only from South Asian countries in the English language were considered for inclusion. Title/ abstract and full-text screening were performed by two reviewers independently (AK, VR). Any discrepancies were resolved through discussion with third reviewer arbitration (RM).

The following PECO* framework explains the eligibility criteria more precisely.

**Table:** 

P	South Asian Countries
E	General Population exposed to covid-19 pandemic, however, it doesn’t refer that such a population is diagnosed with Covid-19
C	Not Applicable
O	Knowledge, Attitude and Practices regarding Covid-19 (KAP)

PECO* (P = Population, E = Exposure, C = Comparison and O = Outcome)

### Data extraction

Extracted data included study details (author, date, study location), study design information (type of design, recruitment method), participant characteristics (target sample, age, gender), measures used, and results of analyses. Studies reporting knowledge or practices in mean or median were not included in quantitative synthesis. Two independent reviewers (AK, MA) carried out the data extraction for each study, and then compared, with discrepancies resolved through discussion.

### Risk of bias assessment

The quality of the included studies was evaluated using a Risk of Bias (ROB) assessment of *Joanna Briggs Institute (JBI) for prevalence data studies tool.* This tool assesses ROB over nine domains, including participant recruitment, sample size and calculation, study subjects, measurement tools and appropriateness of analysis methods (see Table 2). Ratings were made independently by two reviewers (MA, VR) and any conflicts were resolved through third reviewer arbitration (AK). Funnel plots along with egger test value was reported for potential publication bias. Updated searches were screened and extracted by two independent researchers (BA, AK).

### Data synthesis

Narrative synthesis and meta-analysis were utilized for data synthesis. We decided to perform a meta-analysis if at least 3 studies were provided with homogenous characteristics allowing meaningful interpretation of pooled estimates. We set this minimum criterion because our review was based in South Asia, and we wanted to make use of available data. A recent review of the meta-analysis indicated that meta-analysis with three studies is common in medical literature. Studies reporting percentages or observed events were included in the meta-analysis. Overall polled prevalence/ proportions with a 95% confidence interval of knowledge, attitude and practices were generated using double arcsine transformation (Freeman-Tukey transformation) with random effects. To investigate any potential heterogeneity, I^2^ statistics were utilised. Studies reporting overall mean, or medians were not included in the meta-analysis and were summarised in the narrative synthesis [35–43]. In addition, the study participants, outcomes, settings, and findings were also summarised in the narrative synthesis. The study’s characteristics are presented in Table 3.

## Results

### Study characteristics

**Fig. 1:**
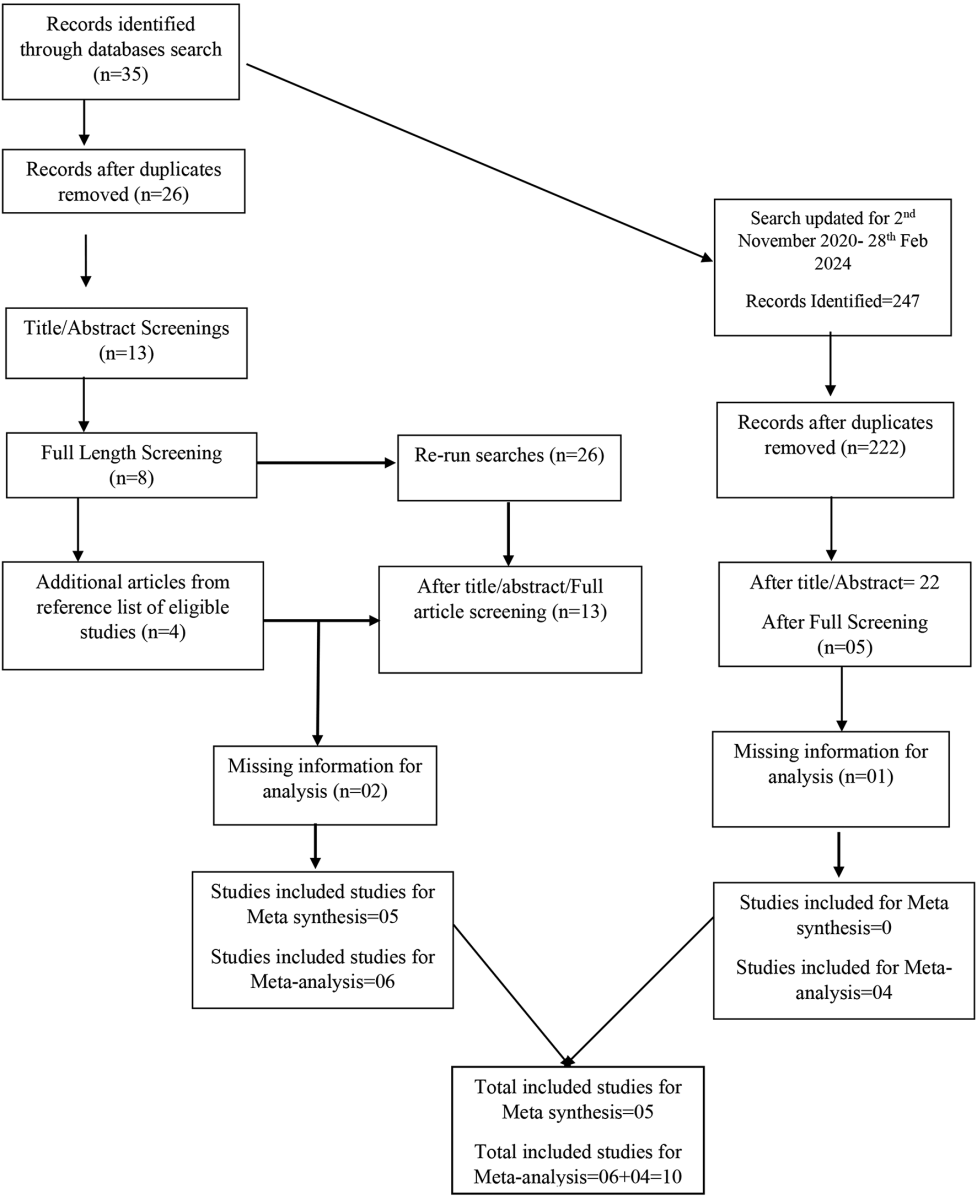
PRISMA flow chart

A total of 282 articles were retrieved from Medline, Cochrane Library, PsychINFO, CINAHL, and Web of Science since inception till 28th Feb, 2024. After duplication removal, 248 articles were included from title and abstract screening, out of which only 18 met the criteria at full length screening. Reference lists of all included articles were also searched for any additional eligible article to be included. 3 articles couldn’t be found in full length as a result a total of 15 articles were included in this review (*See* Fig. 1).

The review provided a narrative synthesis and meta-analysis of the included studies, which is in line with PRISMA guidelines for reporting systematic reviews. The paper screened 282 articles and included 10 studies for meta-analysis with a total of 7,893 participants from South Asian countries, demonstrating a systematic approach. Meta-synthesis was conducted on five studies with 13,476 participants, indicating a qualitative synthesis as recommended by PRISMA.

### Meta-analysis

Most questionnaires and reported statistics in the studies were heterogenous therefore only (*N* = 10) a few studies were included in the quantitative synthesis of frequency rates and total sample size. However, in all included studies separate rates were given for each item targeting any specific knowledge area or practice hence the overall prevalence of knowledge or practice was either meaningless or not reported.

A total of ten studies reported the prevalence of three or more than three of the following: (i) correct knowledge about symptoms (7 studies), (ii) hand washing or use of sanitizer (9 studies), (iii) use of the face mask (9 studies), (iv) herbal and traditional remedies (3 studies) and (v) physical distancing (10 studies). By combining all ten studies, our meta-analysis is based on a total of 7877 participants. Separate pooled prevalence rates were estimated for knowledge of symptoms, handwashing or sanitizing practices, use of masks, any herbal remedies and physical distancing.

**Fig. 2:**
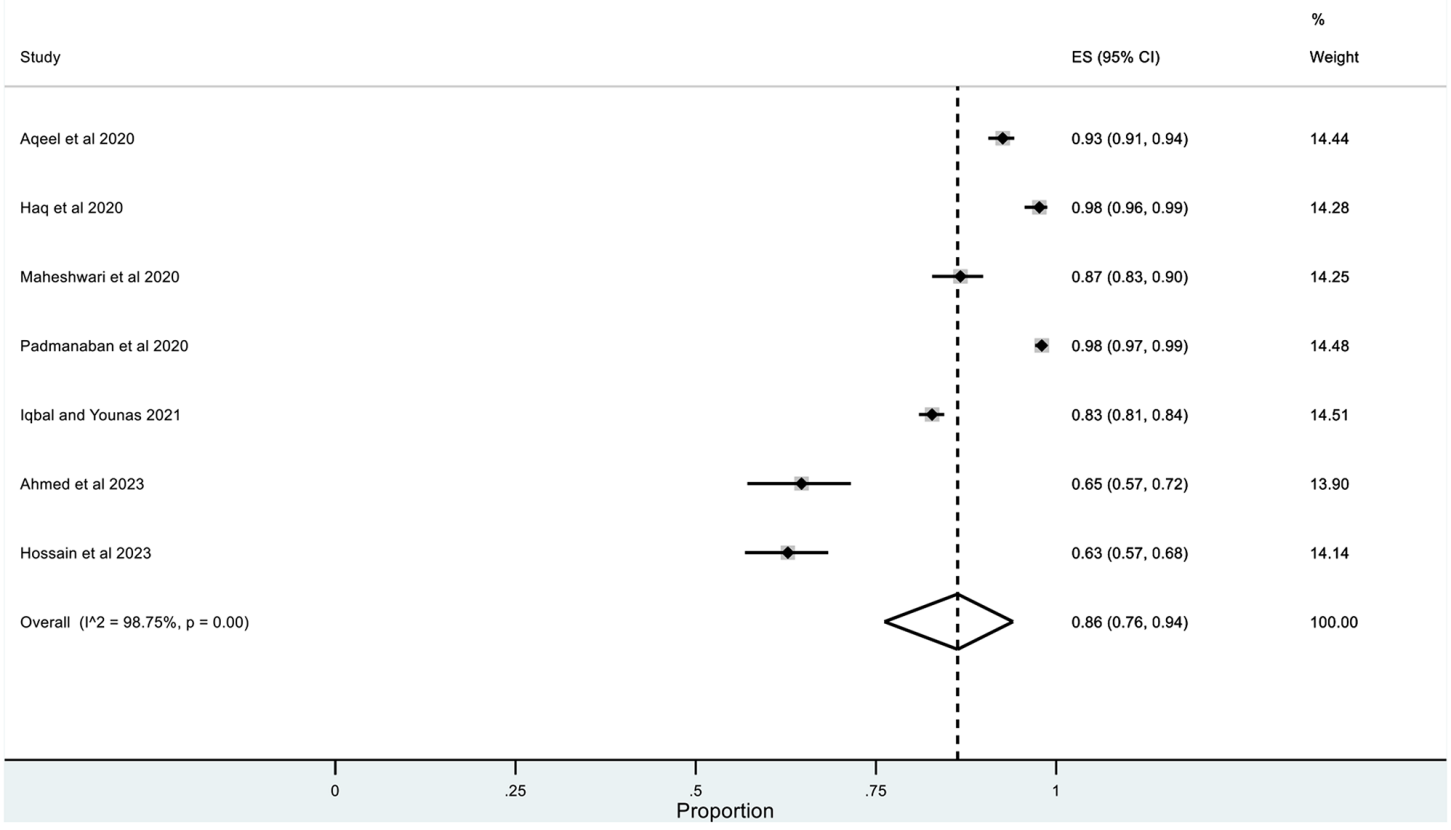
Pooled prevalence of correct knowledge about symptoms

**Fig. 3:**
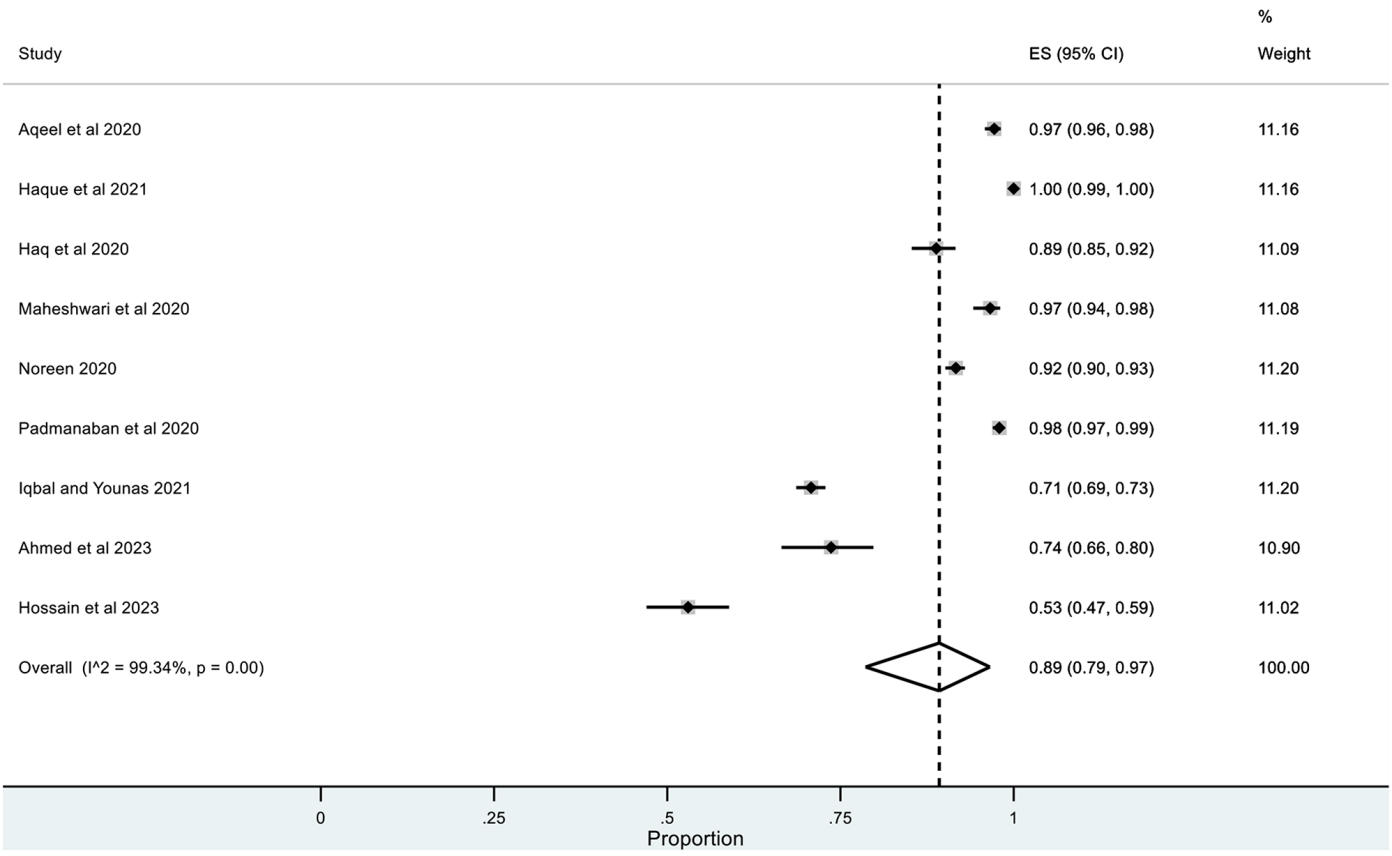
Pooled prevalence of hand washing or use of sanitizers

**Fig. 4:**
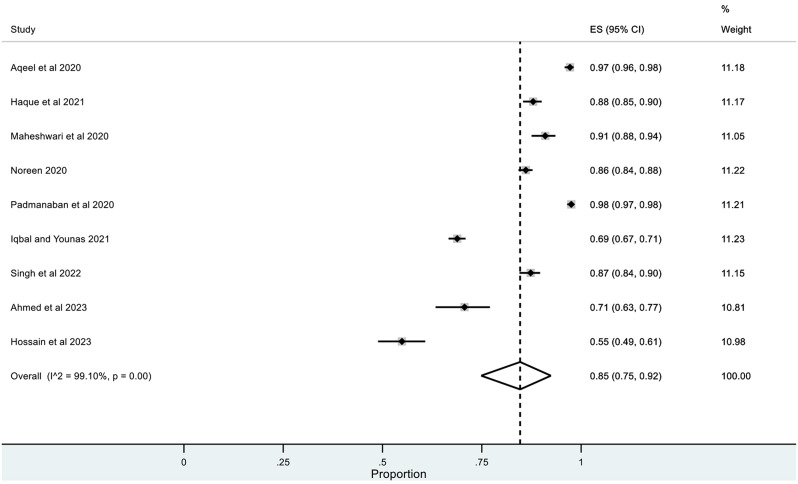
Pooled prevalence of use of face mask

**Fig. 5:**
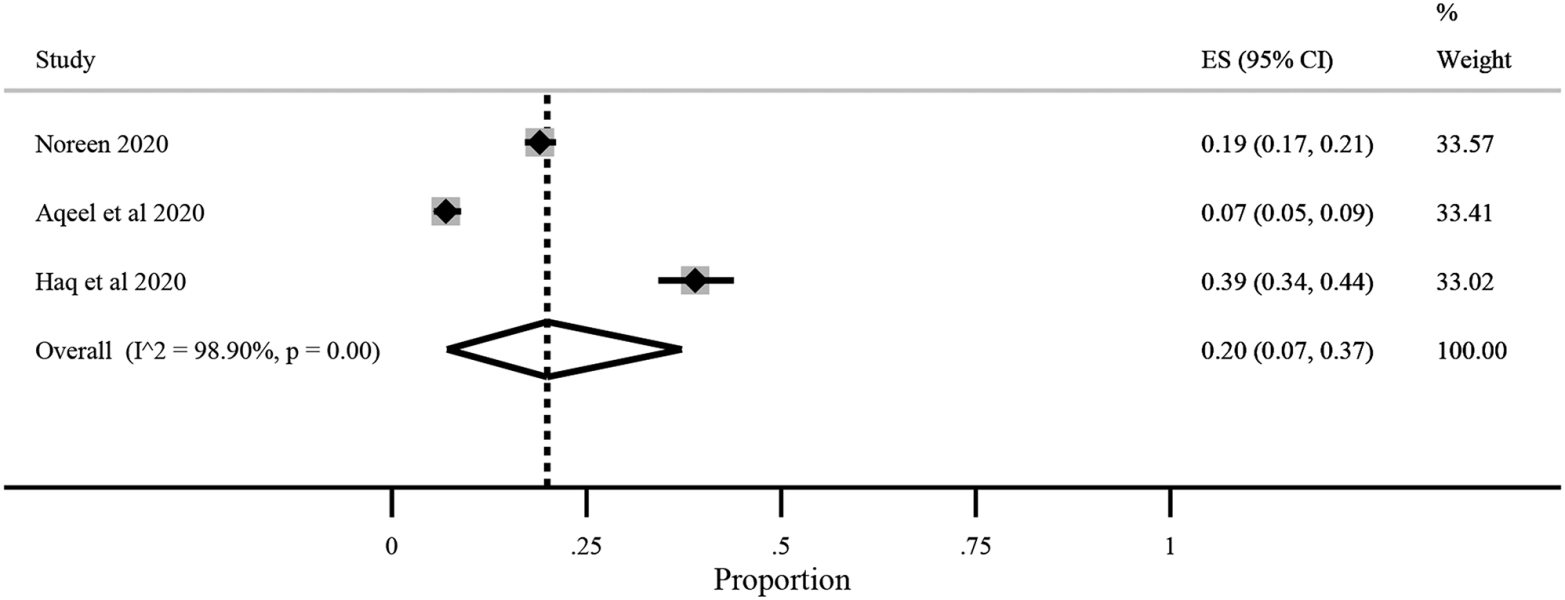
Pooled prevalence of herbal and traditional remedies

**Fig. 6:**
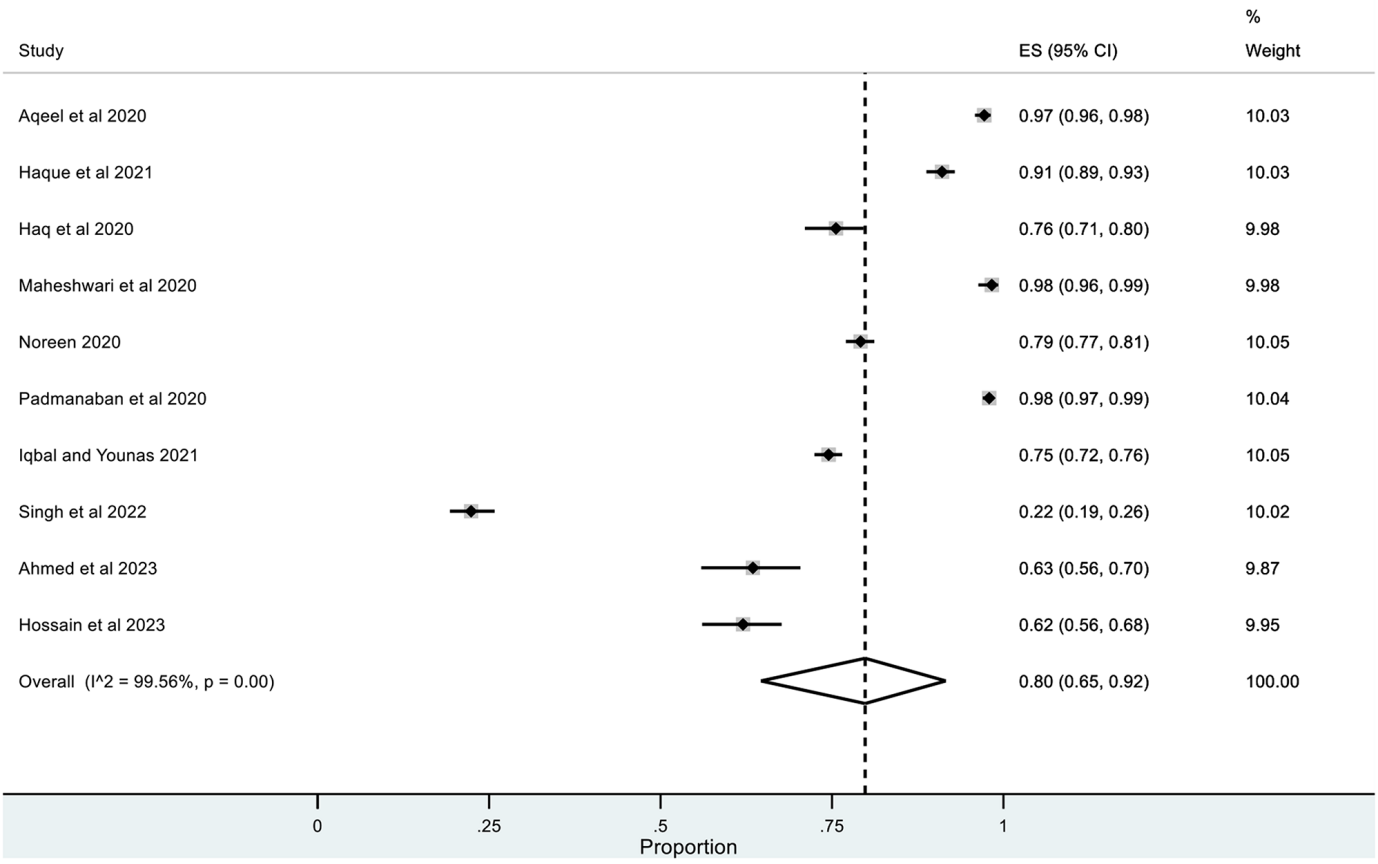
Pooled prevalence of practicing physical distancing or avoiding contact

The pooled prevalence for correct knowledge of symptoms and various practices was generally high with a ceiling effect (except for herbal and traditional remedies) along with high heterogeneity. The overall pooled prevalence for **(1)** correct knowledge of symptoms = 0.86 (95% CI: LLCI = 0.76; ULCI = 0.94) with high heterogeneity (I^2^ = 98.75%) (*See* Figs. 2), **(2)** hand washing or use of sanitizers = 0.89 (95% CI: LLCI = 0.79; ULCI = 0.97) with maximum heterogeneity (I^2^ = 99.34%) (*See* Figs. 3), **(3)** Face Masking = 0.85 (95% CI: LLCI = 0.75; ULCI = 0.92) with maximum heterogeneity (I^2^ = 99.10%) (*See* Figs. 4), **(4)** use of herbal or traditional remedies = 0.20 (95% CI: LLCI = 0.07; ULCI = 0.37) and high heterogeneity (I^2^ = 98.90%) (*See* Figs. 5) and **(5)** physical distancing or avoidance of contact = 0.80 (95% CI: LLCI = 0.65 ULCI = 0.92) and high heterogeneity (I^2^ = 99.56%) (*See* Fig. 6).

All pooled prevalence estimates were associated with very high heterogeneity. Given the limited number of studies, subgroup analysis wasn’t appropriate. However, one of the potential reasons for high heterogeneity was the high sample sizes in studies with lower standard error, increasing the power of test statistics to detect heterogeneity.

**Fig. 7:**
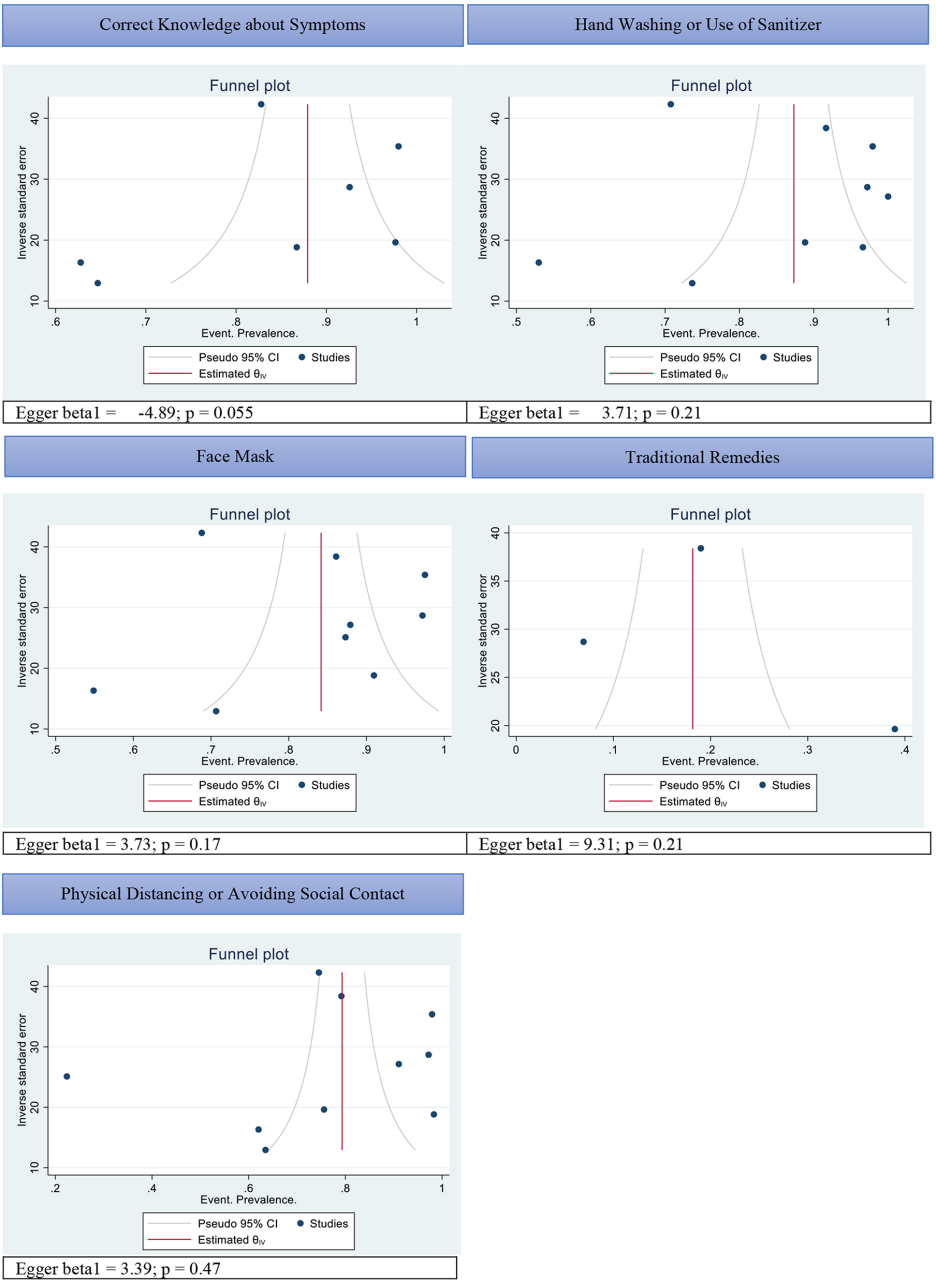
Funnel plot for reporting publication bias

Additionally, the funnel plot indicated a potential risk of bias by demonstrating a diagonal spread of studies either clustered in the lower right or upper left of the plot indicating asymmetry and a potential risk of publication bias (*see* Fig. 7). We also conducted sensitivity analysis by removing studies at high risk of bias, however no major effect on estimates was observed.

### Narrative synthesis

The narrative synthesis included five studies comprising of 13,476 participants from Pakistan, India, Nepal, and Bangladesh. In general, participants exhibited adequate knowledge of COVID-19, positive attitudes toward combating the pandemic and adopted preventative measures, such as social distancing, to avoid the spread of the virus. Notably, participants mentioned the role of religion and culture in coping with the pandemic in seven out of eleven studies. Participants in several studies reported that religious behaviour such as prayer helped them cope with COVID-19 fear [35–37]. Moreover, Haque et al. (2021) reported that people would like religious leaders to help them cope with covid-19 [38]. In addition, several studies reported the use of traditional methods to treat COVID-19 symptoms [16, 37, 38].

### People’s KAP towards COVID-19 and their coping strategies

#### Knowledge

Most studies showed that people were aware of the symptoms and effects of COVID-19, as well as their route of transmission [16, 38–40]. An average of over 70% of the participants were aware of the correct definition of COVID-19 [16, 35–43], apart from the Mamun et al. (2021) where the average correct knowledge score was 57.4% [43]. The respondents knew that COVID-19 is a deadly disease but with early and proper treatment, recovery is possible. Haq et al. (2020) found that the urban population was more knowledgeable than the rural residents [16], whereas Noreen’s (2020) study, reported that females had greater knowledge of COVID-19 compared to males.

#### Attitude

The overall attitude towards COVID-19 was optimistic and positive. Participants believed that the disease is combatable, and it would be controlled eventually [40–42]. In terms of preventing the spread of the virus, a trend towards favouring strict measures was seen [37–39] Although, attitudes towards the pandemic were generally positive, participants also reported a few negative reactions i.e., not taking COVID-19 as a serious problem. Most participants experienced fear at some point during the pandemic [16, 35, 36, 38, 43]. In one study, 63% reported mistrust towards the government in controlling the disease [38]. The virus brought with it great concern for the public as they were at high risk of being infected [38]. Strict measures were taken by the government, for example, travelers had to quarantine, and educational institutes switched to online teaching. The use of print and digital media was reported to spread awareness and news about the virus. The majority had a positive attitude, but some studies showed that females were more hopeful that the spread of COVID-19 can be controlled [41, 42].

#### Practices

*Coping with Covid-19*

Different ways and coping strategies were adopted by people to prevent themselves from getting infected by the virus. Maheshwari et al. (2020) found, isolation and treatment were efficient ways to stop the virus from spreading, and that people should isolate for at least two weeks after coming into contact with an infected person [39]. Most of the studies highlighted that people took greater precautions and hygienic practices such as hand sanitizing/hand washing, face covering and social distancing [16, 39, 40, 42]. Notably, participants mentioned the role of religion and culture in coping with the pandemic in seven out of eleven studies. It was believed that praying and religious activities are most effective in critical, unpleasant circumstances [36, 41]. On the other hand, some believed that there is no specific cure for COVID-19, yet they still turned to medications for recovery [37].

### Risk of bias assessment

All studies with the exception of three provided comprehensive details on participants’ recruitment, sample size, study settings, measurements tools, and data analysis and response rate. Three studies (Khan et al., 2020; Bhawaneshwari et al. 2020; Basu et al.2020) lacked details pertaining to recruitment of participants, study settings, psychometric properties of measurement tools and justification for data analysis and were subsequently removed from synthesis (See Table 2).

## Discussion

The findings of this systematic review and meta-analysis highlighted a high level of heterogeneity in the true knowledge of COVID-19 among included studies. Despite the observed heterogeneity, the findings demonstrated that most of the participants (over 70% in most cases) from included studies possessed correct knowledge of COVID-19. This finding is supported by a recent meta-analysis in China [44] and a cross-sectional survey from Ethiopia [45] that reported over 70% of participants possess adequate knowledgeable about coronavirus. However, it is important to acknowledge the presence of outliers, such as a study [43] reporting a minimum average knowledge level of 57.4%. Similarly, to previous studies, our review also highlighted that participants in urban settings were more knowledgeable about COVID-19 than those living in rural settings [46–49]. This disparity might be due to various factors including poorer access to electricity, mobile networks and digital literacy in rural settings [50, 51]. The limited access to internet connections, digital media platforms and linguistic barriers could be among the factors creating knowledge gap, limiting awareness efforts and impeding the dissemination of information about COVID-19 prevention and treatment in rural areas of South Asia.

Although, information is becoming more accessible online [50], it is not easily accessible in some rural areas, in part due to different education levels and the non-availability of dialect in the local language [51]. The lack of access to the internet, television, or other digital media platforms in rural South Asia can contribute to lower knowledge levels about COVID-19. This knowledge gap may result in limited understanding of preventive measures, symptoms, and treatment options for the disease. Consequently, the overall findings of the study may not accurately represent the knowledge levels of the entire population.

The review also revealed significant gender differences in COVID-19 knowledge, with females having greater knowledge of COVID-19. Similarly, Sultana et al. (2022) reported significant gender differences regarding knowledge of COVID-19 where females had more knowledge. Social media use in females [52] as a significant link between sources of information and knowledge has been highlighted by various studies [17, 53, 54]. Social media platforms became crucial sources of information during the pandemic therefore; this increased exposure may lead to greater engagement with COVID-19 related content leading to increased knowledge levels. Further, in most cultures, women generally have the role of looking after the family and the household. Since women often play a central role in healthcare decisions for their family, this may serve as a motivation to stay informed and seek out reliable information. More time at home may also provide more opportunities for social media use which as a result may raise awareness and better knowledge [55]. In contrast to this, male participants from a study [56] conducted in Lebanon scored high on some questions about knowledge of COVID-19 including questions related to the cause and symptoms as compared to females. This can be explained in light of previous research from various countries consistently reporting an advantage of males over females in general knowledge as well as biologically differentiated interests [57].

Although the review unveiled a positive attitude of participants towards measures to reduce the spread of SARS-CoV-2, there were also some instances of negative attitude. Participants reported fear [16, 35, 36, 38, 58], and a high risk of being infected [38]. In a previous study [59], participants across different cohorts among Asian countries were found to be more fearful. This is important as such fear has been linked to mental health difficulties such as depression, anxiety, and stress [1]. The review also reported mistrust towards the government in controlling the disease [38] however the role of important predictive factors such as the adoption of health behaviours, prosocial behaviours [59], education, and media freedom [60] has not been explored [60].

Regarding coping strategies, along with preventive measures against COVID-19 [16, 39, 40, 42] participants also highlighted the role of religious coping such as praying and religious activities to combat COVID-19 [36, 41]. Similarly, Bentzen’s study [61] which used daily searched data records of Google from 95 countries and demonstrated increased Google searches for prayer to the highest ever recorded level during the COVID-19 crisis. Additionally, the study established that more than half of the world population had prayed to end the coronavirus. Many people have strong religious beliefs, providing them an anchor and consequently helping people cope. Religious coping might act as a potential tool for managing stress during illnesses and challenging situations like the COVID-19 pandemic, as well as improving physical and mental health outcomes [62–64].

The review has several strengths and highlighted differences in KAP among genders as well as differences in information distribution in rural setting and urban settings and different cultures. The review enhanced our understanding of the socio-cultural influences on pandemic responses. The positive attitudes towards measures and use of religious coping strategies across studies indicated that SAs possess a strong societal willingness to engage in preventive measures. These findings could be useful to tailor public health interventions for individuals in diverse settings. However, it is important to acknowledge the limitations of the review such as heterogeneity and potential biases among included studies, which may affect the generalizability of findings. Future research should address these limitations and further explore the socio-cultural determinants of COVID-19 knowledge and behaviour. Addressing these gaps could enhance the effectiveness and cultural sensitivity of preventive interventions.

## Conclusion

The insights gained from this review offer valuable guidance for future pandemic preparedness and response efforts. The review emphasizes the complex socio-cultural factors that influence responses to the pandemic, including risk perceptions and coping strategies. Policymakers, healthcare professionals along with other potential stakeholders such as community representatives and gatekeepers can create more effective and targeted interventions to improve community resilience and promote public health and well-being by customizing interventions to address differences in individual and cultural KAP related to the future pandemics.

**Table 1:** Data Synthesis

Searches	Results	Type	Actions	Annotations
	1	(perception or Knowledge or Information or Attitude* or Awareness or Practices or Opinions or Beliefs).mp. [mp = ti, ot, ab, sh, hw, kw, tn, dm, mf, dv, fx, dq, tc, id, tm, mh, nm, kf, ox, px, rx, an, ui, sy]	8,485,050	Advanced
	2	Religio*.mp. [mp = ti, ot, ab, sh, hw, kw, tn, dm, mf, dv, fx, dq, tc, id, tm, mh, nm, kf, ox, px, rx, an, ui, sy]	248,453	Advanced
	3	(COVID-19 or COVID or Coronavirus or SARS-CoV-2).mp. [mp = ti, ot, ab, sh, hw, kw, tn, dm, mf, dv, fx, dq, tc, id, tm, mh, nm, kf, ox, px, rx, an, ui, sy]	277,165	Advanced
	4	(Pakistan or India or Bangladesh or Sri Lanka or Nepal or Bhutan or Maldives or Afghanistan or South Asia*).mp. [mp = ti, ot, ab, sh, hw, kw, tn, dm, mf, dv, fx, dq, tc, id, tm, mh, nm, kf, ox, px, rx, an, ui, sy]	585,316	Advanced
	5	1 and 2 and 3 and 4	35	Advanced
	6	remove duplicates from 5	26	Advanced

**Table 2:** Risk of bias assessment

Study No.	Authors and Year	1) Was the sample representative of the target population?	2) Were study participants recruited in an appropriate way?	3) Was the sample size adequate?	4) Were the study subjects and the setting described in detail?	5) Was the data analysis conducted with sufficient coverage of the identified sample?	6) Were objective, standard criteria used for the measurement of the condition?	7) Was the condition measured reliably?	8) Was there appropriate statistical analysis?	9) Was the response rate adequate, and if not, was the low response rate managed appropriately?	Overall Appraisal (Include/ Exclude/ seek futher info)
1	Hossain et al., 2020	Yes	Yes	Yes	Yes	Yes	Yes	Yes	Yes	Yes	Include
2	Yasin S.A., 2020	Yes	Yes	Yes	Yes	Yes	Yes	Yes	Yes	Yes	Include
3	Khan et al., 2020	No	Unclear	Yes	No	Unclear	Unclear	Unclear	Unclear	No	Exclude
4	Iqbal M.A. & Younus M.Z, 2021	Yes	Yes	Yes	Yes	Yes	Yes	Yes	Yes	Yes	Include
5	Singh et al., 2020	Yes	Yes	Yes	Yes	Yes	Yes	Yes	Yes	Yes	Include
6	Maheshwari et al., 2022	Yes	Yes	Yes	Yes	Yes	Yes	Yes	Yes	Yes	Include
7	Mamun et al., 2021	Yes	Yes	Yes	Yes	Yes	Yes	Yes	Yes	Yes	Include
8	Haq et al., 2020	Yes	Yes	Yes	Yes	Yes	Yes	Yes	Yes	Yes	Include
9	Bhawaneshwari et al. 2020	No	Unclear	Yes	Unclear	Unclear	Unclear	Unclear	Unclear		Exclude
10	Haque et al. 2021	Yes	Yes	Yes	Yes	Yes	Yes	Yes	Yes	Yes	Include
11	George et al. 2020	Yes	Yes	Yes	Yes	Yes	Yes	Yes	Yes	Yes	Include
12	Noreen et al. 2020	Yes	Yes	Yes	Yes	Yes	Yes	Yes	Yes	Yes	Include
13	Aqeel et al. 2020	Yes	Yes	Yes	Yes	Yes	Unclear	Yes	Yes	Yes	Include
14	Basu et al.2020	No	No	Unclear	No	Yes	Yes	Unclear	Unclear	Unclear	Exclude
15	Hossain et al. 2023	Unclear	Yes	Yes	Yes	Yes	Yes	Unclear	Yes	Unclear	Include
16	Ahmed et al. 2023	Unclear	Yes	Unclear	Yes	Yes	Yes	Yes	Yes	Unclear	Include
17	Padmanaban et al. 2022	Yes	Yes	Yes	Yes	Yes	Yes	Unclear	Yes	Unclear	Include
18	Singh et al. 2022	Yes	Yes	Yes	Yes	Yes	Yes	Yes	Yes	Yes	Include

**Table 3:** Study characteristics of included articles

Author, Year, Country	Study Design	Participant characteristics			Risk perception (KAP) towards COVID-19			Cultural-religious belief	Coping with COVID-19
		Sample size	Age	Gender	Knowledge	Attitudes	Practices		
Haque et al., 2021, Pakistan	Cross-sectional	737 literate participants	Median = 32 ± 8	52% male, 48% female	Correct definition (85%), knowledge for route of transmission (92%), knowledge of a curative treatment (21%), knowledge of vaccine (8%)	Concern (82%), mistrust of government (63%), favour strict measure,	Handwashing (100%), avoiding contact (91%), covering coughs (89%), wearing masks (88%), use of disinfectant (77%)	Virus disappears in hot weather (59%), hot lemon/ginger water preventative (57%/32%), herbal supplements/raw onion preventative (38%/29%)	Lead responsibility: health authorities (35%), armed forces (29%), government (26%), religious leaders (10%)
Yasin, 2020, Pakistan	Cross-sectional	317 non-educated Pakistani citizens	Range = 18 to 50+, majority 23–38 (40.10%)	121 male, 196 female	NM	High levels of fear (male = 59.50%; female = 63.26%), fear in accordance to society gender roles	NM	Religion protective against fear	NM
George, 2020, India	Cross-sectional cohort study	64 healthcare team of doctors, nurses, paramedical and support staff	Mean = 34.6, SD = 10.7	24 male, 40 female	Participants are healthcare professionals	Fear experienced at some point of time (75%),	NM	Prayer as the most critical thing to overcome covid-19 (40.6%)	Distracting with hobbies (20.3%), spending time with family (39.1%)
Noreen, 2020, Pakistan	Cross-sectional cohort study	1474 medical students	NM	576 male, 898 female	Participants are medical students, adequate knowledge (71.7%)	Positive attitudes (92.5%), covid has an impact on wellbeing (69%)	Good practice (95.4%)	Become more religious (26%)	NM
Aqeel et al., 2020, India	Cross-sectional	823 General Population	Mean = 38.3, SD = 1.95	469 male, 353 female	Knowledge about the symptom (92.58%),	Covid as a social stigma (73.74%), Believe in lockdown measures (76.79%),	Adherence to health steps (97.2%),	Performing prayer and taking herbal medication	Doctors as the best person to take suggestion (69.62%)
Haq et al., 2020, Pakistan	Cross-sectional	401 General Public	63.6% < 30 years	52.3% male, 47.7% female	Generally good knowledge (Urban more knowledgeable than rural)	Perceive high level of risk (55% urban, 50% rural)	Hygiene behaviour, avoidant behaviour	Using traditional methods to save themselves (57.5% urban, 64.9% rural), greeting other people in traditional ways (28.6% rural, 21% urban)	Avoiding public transport (95.3% urban, 86.9% rural)
Maheshwari et al., 2020, India	Cross-sectional cohort study	354 Medical Students	89.8% 18–23 years	178 male, 176 female	Participants are medical students, correct knowledge (86.7%)	Support lockdown measures (94.1%), in favour of ‘Janta curfew’ (75%),	Avoid unnecessary travel or outing (98.6%), hygiene behaviour (96.9%)	Participants belonging to the Hinde religion had more knowledge, more positive attitude and more precaution practices than Muslim and other religions (non-significant)	NM
Hossain et al., 2020, Bangladesh	Cross-sectional	2157 respondents from Bangaldesh	Mean = 33.48, SD = 14.65	1166 male, 991 female	Average knowledge scores 8.71/12, knowledge corrected with education level	Belief in control (62%), Positive attitudes correlated with education level	Wearing masks (83.7%), avoiding crowds (75.4%)	NM	NM
Iqbal and Younas, 2021, Pakistan	Cross-sectional cohort study	1789 well-educated individuals from Pakistan Universities	Mean = 23.4, SD = 8.23	840 male, 949 female	Participants are educated, average knowledge score 9.6/13	Belief in control (58%),	Wearing face masks (69%), avoiding social/festive event (75%)	Belief that covid-19 is the result of an international conspiracy (66% agreed or unsure)	Choose to stay at home without medical advice if exhibiting symptoms (53%)
Mamun et al., 2021, Bangladesh	Cross-sectional	10,067 individuals covering all 64 districts in Bangladesh	Mean = 29.9, SD = 9.6	56.1% male, 58.4% female	Average knowledge scores 11.48/20	Average scores for fear of covid-19 21.3/35	Average preventative behaviour scores 4.23/20	NM	NM
Singh, 2020, Nepal	Cross-sectional	871 General Adult Population	Mean = 26.4, SD = 6.3	40.4% male, 59.6% female	Median knowledge scores 10 out of 13	Belief in personal hyenine and social distancing would prevent the spread of virus (96.1%)	NM	NM	NM
Hossain, 2023, Bangladesh	Interview-based cross-sectional study	266 rural adolescents	Adolescents aged 10–19 years	55.6% female	• Mean knowledge score = 7.15 (out of 12) • 11% had adequate knowledge • 74% had moderate knowledge • 15% had inadequate knowledge	• Mean attitude score = 10.5 (out of 16) • 27% had positive attitude • 58% were neutral	• Mean practice score = 8.78 (out of 14) • 31% had good practices • Preventive practices like hand washing (53% always did), mask wearing (54.9% always did)	Adolescents from Islamic religion demonstrated higher knowledge and positive attitude	• Mass media like TV and newspapers was the main source (37.6%) of COVID-19 information • Factors like religion, education level, family income associated with knowledge, attitudes and practices
Ahmed, 2023, Bangladesh	Cross-sectional survey study	167 undergraduate university students	Not provided	64.1% male, 35.9% female	• Most students had heard about COVID-19 from various sources like social media, news media, international organizations, friends/family • Many knew about transmission, symptoms, isolation needs, handwashing, masking and need for vaccine • However, around 69% did not think children and adults were affected by COVID-19 • Females and social science students had higher knowledge scores	• Favorable attitudes towards social distancing, lockdowns, self-protection against COVID-19 • Hindu students and urban residents had more favorable attitudes	• Higher practices like masking, sanitizer use, avoiding handshakes and crowded places among later semester students • Urban residents had higher practice scores than rural	Around 31% strongly agreed that COVID-19 would not spread in religious crowds	Major impacts mentioned were fear of getting sick, social distancing, job insecurity, university closures, quarantine
Padmanaban, 2022, India	Cross-sectional survey	1252 higher education students	59.3% <= 21 years, 40.7% > 21 years	32% male, 68% female	• 65.5% had high level of knowledge about COVID-19 • 34.5% had low or moderate knowledge • Overall 71% correct answer rate on knowledge test	• 71% had positive attitude towards COVID-19 • 18% had neutral attitude • 11% had negative attitude • Overall 86% positive attitude score	• 66.7% exhibited desirable practices to prevent COVID-19 • 18.7% had neutral practices • 14.5% had undesirable practices • Overall 86% desirable practice score	24.7% believed COVID-19 is a sinful disease	• Social media (81%) was the main source of information about COVID-19 • Only 25% relied on authentic sources
Singh, 2022, India	Observational study	630 blood donors	• 55.1% aged 18–29 years • 38.3% aged 30–49 years • 6.7% aged 50–65 years	• 93.2% male • 6.8% female	• Knowledge scores were significantly associated with marital status, education, and occupation • Higher knowledge scores in those with master’s degree education	• 57.3% had positive attitude that COVID-19 will be controlled • 75.9% agreed that removing fear from blood donors can increase blood donations	• 77.6% visited crowded places during pandemic • 87.3% wore masks while donating blood	No significant difference in knowledge based on religion (Hindu vs. Muslim)	Suggests improving knowledge through health education platforms to increase blood donations during pandemic

## Data Availability

The data and materials used in this systematic review are available upon request by email to the corresponding author m.rakhshi@ucl.ac.uk.
